# Semaglutide, type 2 diabetes, and the risk of nonarteritic anterior ischemic optic neuropathy

**DOI:** 10.1186/s40942-024-00622-9

**Published:** 2025-01-22

**Authors:** Fernando K. Malerbi, Marcello C. Bertoluci

**Affiliations:** 1 Federal University of São Paulo, São Paulo, Brazil; 2 Brazilian Diabetes Society, São Paulo, Brazil; 3 Federal University of Rio Grande do Sul, Porto Alegre, Brazil

**Keywords:** Type 2 diabetes, Semaglutide, Nonarteritic anterior ischemic optic neuropathy

## Abstract

In the last months, conflicting evidence on a possible association between the use of semaglutide and incident nonarteritic anterior ischemic optic neuropathy (NAION) has emerged. A recently published study, which evaluated all patients with type 2 diabetes in Denmark, has shown with robustness that once-weekly semaglutide doubles the five-year risk of NAION. In this comment, the new evidence is discussed, along with practical implications for type 2 diabetes patients. The possibility of ophthalmological evaluation regarding optic disc morphology is suggested, before initiation of semaglutide treatment or, for those patients already under treatment, during a follow-up ophthalmological visit. If a disc-at-risk pattern is detected, such information could be brought to the attention of the attending clinician involved with diabetes control and discussed with patients for a shared decision-making approach. A new risk-benefit discussion weighing the undoubted benefits of semaglutide in reducing cardiovascular mortality and cardiovascular events, heart failure hospitalization, and renal protection must be started and carefully balanced against a rare but devastating condition such as NAION.

## Background

A growing discussion regarding the relationship between the use of the glucagon-like peptide-1 receptor agonist (GLP-1 RA) semaglutide and the rare but devastating nonarteritic anterior ischemic optic neuropathy (NAION) is emerging in the global scenario of type 2 diabetes and obesity treatment, launching concerns on the use of one of the most popular prescribed drug, after conflicting conclusions from recent studies were published.

## Main text

In an article published in July 2024, Hathaway and colleagues suggested an association between the use of semaglutide and the diagnosis of NAION, a severe ocular condition which potentially causes significant visual impairment [1]. Following that publication, the issue became a hot topic both in endocrinology and ophthalmology forums, as the drug has been increasingly prescribed since its approval by regulatory agencies; a study on national trends in prescription drug expenditures in the USA concluded that semaglutide was the top drug in 2023 [2]. The advent of GLP1-RA has undoubtedly revolutionized the care of type 2 diabetes (T2D), due to the high efficacy in improving levels of glycated hemoglobin A1C and fasting glucose, reducing body weight, improving quality of life, and mainly due to the reduction in morbidity and mortality of major adverse cardiovascular events and heart failure [3].

Even though many repercussions ensued, recommendations for new semaglutide prescription or the management of T2D patients already in use of the drug did not change at that time: the authors themselves stated that their results should be considered with caution, and ensuing comments mentioned that the potential risk of NAION should not deter the directed use of GLP-1 RA in T2D at that time, as more evidence was needed to confirm or refute such association [1, 3]. Among the reasons for such caution were mainly the large number of people globally in use of GLP-1RA, while NAION is undoubtedly a rare outcome, as well as the retrospective, single-center design of that study; Hathaway and colleagues suggested that a much larger, retrospective, multicenter population-based cohort study would help to confirm, refute or refine their findings [1].

More recently, Chou and colleagues published the results from a retrospective cohort, using medical records from the TriNetX Analytics Network, a global platform that includes electronic medical records from 160 healthcare organizations across 21 countries, encompassing around 200 million patients; the authors analyzed data from 37,245 individuals in the T2D-only cohort, 138,391 in the obesity-only cohort, and 64,989 in the T2D with obesity cohort, to search for an association between semaglutide and NAION. Groups were well-balanced through a 1:1 propensity score matching, and a sensitivity analysis was performed. The effect of semaglutide was compared with patients not using GLP-1 RA, and NAION was evaluated as the primary outcome at one, two, and three years of follow-up. The results indicated that the administration of semaglutide was not associated with the development of NAION in either group [4].

In December 2024, another study raised new concerns on this theme. A novel study by Grauslund and colleagues, just published in this journal, reinforces the association of once-weekly semaglutide use by T2D patients and NAION. This was an impressively large study, using data from the Danish National Patient Registry and the Danish National Prescription Registry, consisting of a prospective cohort that evaluated the whole population of Denmark with T2D, including 424,152 persons exposed (*n* = 106,454) or unexposed (*n* = 317,698) to once-weekly semaglutide [5]. The authors defined T2D diagnosis by combining International Classification of Disease codes, once-weekly semaglutide exposure by Danish National Prescription Registry coding, and NAION outcome by its diagnostic code in the Danish National Patient Registry.

Among 1,915,1120 person-years of observation, 218 individuals developed NAION, 67 in the exposure group and 151 in the non-exposure group (calculated risk of 0.228 versus 0.093 per person-years, respectively). The authors concluded that using once-weekly semaglutide more than doubled the risk of NAION during five years of observation. Such association was independent of several covariates also evaluated by the authors, including age, duration of diabetes, hemoglobin A1C, estimated glomerular filtration rate, cardiovascular disease, use of insulin, use of cholesterol-lowering medicine, use of blood pressure lowering medicine and diabetic retinopathy [5].

An intriguing information brought by Grauslund and colleagues is related to the historical trend of NAION in Denmark: they report that both the annual number of first-time NAION episodes and the rate of prevalent T2D among patients with newly diagnosed NAION episodes have risen significantly in the period encompassing the years 2019–2023, in comparison to the 2003–2018 period; this surge coincided with the availability of semaglutide in the Danish market, starting in December 2018 [5].

Contrary to the study by Hathaway and colleagues, which pointed to the highest risk of NAION within the first year following the prescription of semaglutide, the study conducted by Grauslund and colleagues did not find any high-risk window between exposure and outcome. The median time from the redemption of the first prescription to NAION was 22.2 months, and the onset of the event was evenly distributed within the entire five-year observation period [5].

Notably, the authors themselves mention several limitations to their study that may impact the conclusions, including the lack of access to relevant clinical data such as smoking, blood pressure, or body mass index; the lack of access to ophthalmic examinations; the lack of information on patient’s adherence to drug use; and the impossibility of examining the duration of exposure to the drug in the analyses. They clearly state that their study cannot claim a causal relationship between exposure and outcome. They also emphasize that the overall risk of NAION was still low, with an incident rate of 0.228 per 1000 persons-years for persons with T2D [5].

Even though NAION is considered a rare disease, it is the most common acute optic neuropathy in adults. It has no known treatment, presenting as an acute spontaneous symptomatic event that occurs unilaterally, the extent of visual loss varying widely, potentially evolving to almost complete blindness [6]. In addition to several potential systemic risk factors, there are relatively established morphological patterns in the optic disc anatomy associated with increased NAION risk, such as a small cup-to-disc ratio and/ or the presence of optic disc drusen [6, 7]. Such patterns could generally be detected by the ophthalmologist who is already following a T2D patient for diabetic retinopathy detection or treatment.

Effective treatments for T2D are of utmost importance in a diabetes epidemic scenario [8]; the advent of GLP1 – RA has revolutionized T2D treatment, improving the quality of life of T2D patients and preventing serious systemic outcomes. While the possible relation between semaglutide and the worsening of diabetic retinopathy is still the object of study, with an ongoing clinical trial designed to shed light on this issue [9], and even though the potential association between semaglutide use and NAION is not definitive, patients, clinicians, and ophthalmologists should be aware of such possible association. Future prospective studies could be challenging to perform due to the rare nature of the outcome; nevertheless, new observational studies could contribute to a better understanding in the future, as well as studies that try to identify subgroups at higher risk of developing NAION. A postmarketing analysis of all GLP-1 RA, including different dosing and administration routes, could also be helpful.

In the current state of knowledge, however, due to the irreversible nature of NAION, and since the exact pathogenesis of NAION is still unknown, it makes sense that, in addition to looking for diabetic retinopathy, ophthalmologists also begin to turn their attention to the optic disc pattern. Such evaluation of the optic disc could take place before initiation of semaglutide treatment or, for those patients already under treatment, during a follow-up ophthalmological visit. If a disc-at-risk pattern is detected, such information could be brought to the attention of the attending clinician involved with diabetes control and discussed with patients for a shared decision-making approach and the weighing of risks versus benefits.

Grauslund et al. should be praised for their study, which analyses the incidence of NAION in T2D patients who were prescribed once-weekly semaglutide; a great knowledge gap still exists concerning semaglutide for other clinical indications, such as the treatment of obesity. Further studies are needed to evaluate the potential association of NAION and semaglutide use for non-diabetic patients.

## Conclusions

In conclusion, a new risk-benefit discussion weighing the undoubted benefits of semaglutide in reducing cardiovascular mortality and cardiovascular events, heart failure hospitalization, and renal protection must be started and carefully balanced against a rare but devastating condition such as NAION.

## Data Availability

No datasets were generated or analysed during the current study.

## Abbreviations

NAION: Nonarteritic anterior ischemic optic neuropathy
GLP-1 RA: Glucagon-like peptide-1 receptor agonist
T2D: Type 2 diabetes
